# Proof of Concept for a Digital Framework to Support a Shared Agenda at Surgical Ward Rounds: Participatory Design Study

**DOI:** 10.2196/69679

**Published:** 2025-06-19

**Authors:** Helle Poulsen, Jane Clemensen, Jette Ammentorp, Poul-Erik Kofoed, Maiken Wolderslund

**Affiliations:** 1 Department of Surgery, Lillebaelt Hospital, University Hospital of Southern Denmark, Sygehusvej 24, Kolding, DK-6000, Denmark, 45 76362665; 2 Department of Regional Health Research, Faculty of Health Sciences, University of Southern Denmark, Odense, Denmark; 3 OPEN, Open Patient data Explorative Network, Odense University Hospital, Odense, Denmark; 4 Hans Christian Andersen Children’s Hospital, Odense University Hospital, Odense, Denmark; 5 Centre for Innovative Medical Technology, Odense University Hospital, Odense, Denmark; 6 Department of Clinical Research, Faculty of Health Sciences, University of Southern Denmark, Odense, Denmark; 7 Centre for Research in Patient Communication, Odense University Hospital, Odense, Denmark; 8 Department of Paediatrics and Adolescent Medicine, Lillebaelt Hospital, University Hospital of Southern Denmark, Kolding, Denmark; 9 Danish Centre for Clinical Artificial Intelligence (CAI-X), University of Southern Denmark and Odense University Hospital, Odense, Denmark

**Keywords:** surgical ward rounds, structured interprofessional bedside rounds, digital technologies, logistics system, patient participation, family involvement, mobile health app

## Abstract

**Background:**

Surgical ward rounds (SWRs) are often unstructured and deprioritized compared to traditional surgical tasks, leading to limited interdisciplinary collaboration, unprepared patients, and low family attendance.

**Objective:**

This study aims to co-design and develop a digital framework to facilitate a shared agenda for SWRs, ensuring all core participants can attend and participate effectively.

**Methods:**

Participatory design (PD) methodologies were used, using user-engaging activities within an iterative process. A multidisciplinary team, including patients, relatives, health care providers, technology designers, and researchers, collaborated in workshops and testing to translate user needs into prototypes of technologies consisting of the digital framework.

**Results:**

A logistics system was developed for nurses to prebook the SWRs in designated time slots, enabling them to prepare relevant data and partake in the dialogue with patients. In addition, a mobile health (mHealth) app displayed the schedule for patients and relatives, helping them to participate and prepare questions in advance. Multiple iterations ensured that the digital framework met user needs and was feasible for clinical practice.

**Conclusions:**

Our findings underscore the importance of collaboration between users and technology designers in developing digital health technologies. Engaging the users helped identify technical and organizational constraints that needed to be addressed to integrate the digital framework into clinical settings.

## Introduction

### Background

Surgical ward rounds (SWRs) are crucial for the communication between patients, their families, surgeons, and the care team, providing opportunities for high-quality, collaborative, and person-centered care planning [1,2]. Nevertheless, research demonstrates that SWRs are often unstructured and deprioritized compared to other surgical tasks, compromising interdisciplinary collaboration, patient and family involvement, and patient safety [3-6]. Due to the senior surgeons’ numerous competing commitments, junior doctors often lead the SWRs with minimal learning opportunities and supervision, affecting round quality, efficiency, and structure [7,8]. The unpredictable nature of the SWRs results in the bedside nurses being unprepared and limits their access to attend. Consequently, it hampers their ability to properly contribute with relevant patient information and follow-up [9-13]. Accordingly, patients and their relatives experience the SWRs as disruptive, short, and with a narrow medical focus, making it difficult for them to participate actively. Patients are often unprepared for the SWRs and can not distinguish between the many health care providers attending the room [14]. Consequently, they are not always aware of the SWRs taking place [15-19]. Due to the lack of planning, the relatives seldom have the chance to attend. As a result, they feel uninvolved and lack information [20,21]. Altogether, existing research indicates that the timing and agenda for the SWRs are primarily set by the doctors, making nurses, patients, and relatives merely passive recipients of treatment decisions and care plans. A central part of person-centered health care communication is identifying issues the patient wishes to address, thereby negotiating a shared agenda for the encounter. Furthermore, a mutual plan of action should be negotiated by involving the patients and relatives in decision-making [22,23]. For this to happen, the participants must be well prepared and given the opportunity to partake. However, the existing organizational structure in the surgical wards seems to hinder the chances of initiating a truly person-centered dialogue. Several studies indicate that implementing a structured approach by informing patients of the timing of the SWRs enhances their readiness for participation and facilitates family attendance. Furthermore, prioritizing a dedicated time for SWRs would enable nurses to schedule their day more effectively, ensuring they are prepared and can attend [15,16,21,24,25]. Building on this previous knowledge, our study explores how such structured approaches can be adapted and implemented within the specific organizational context of SWRs. Digital technologies have been suggested to support nurses, patients, and relatives to partake in ward rounds, eg, by notifying nurses and patients via electronic devices [26-30], mobile health (mHealth) apps [31-33], and video communication with relatives [34-38]. Patients and health care providers recognize the benefits of these digital technologies [14]. However, existing solutions are fragmented, typically targeting only a single participant group, and their adoption is limited by user reluctance, as well as technical and organizational barriers [26-28,31,33,37]. To unlock their full potential, digital technologies must be integrated into more innovative, user-centered designs that align with the needs of key participants and the clinical settings in which they are intended to be used [32]. A suitable method for developing digital technologies that meets the needs of both patients, relatives, and health care providers is participatory design (PD). Central to PD is mutual learning, aiming to balance the power between users and technology designers through knowledge sharing. Researchers and designers require a deep understanding of the needs, clinical context, and experiences of the users, while users benefit from the technological knowledge of the designers. This collaborative and democratic approach empowers users to influence the design of digital technologies affecting their lives [39].

### Objective

This study aims to co-design and develop a unified digital framework to ensure that all core participants can actively engage in and contribute to the agenda and decisions made at SWRs. We define a digital framework as a structured system that supports communication and collaboration among health care providers, patients, and relatives, with intentional coordination of both human and technical components.

## Methods

### Study Design

**Figure 1.**
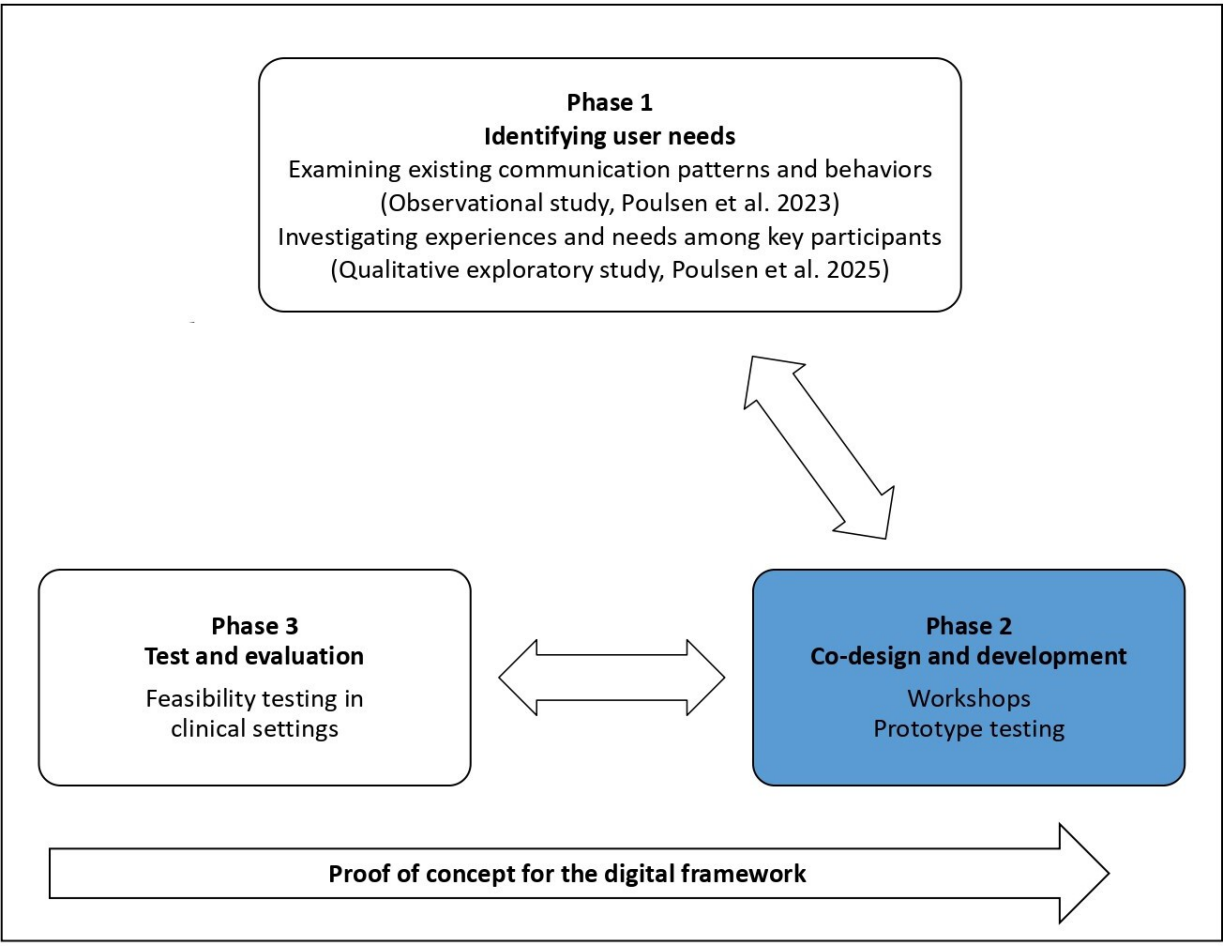
The 3 phases of the digital framework design and development [14,20].

In health research, PD studies typically adopt an iterative, phase-driven approach, beginning with identifying user needs, followed by prototype design and development, and concluding with pilot testing and evaluation [40,41]. In Phase 1, we have investigated existing communication patterns and behaviors during SWRs as well as experiences and needs among key participants. The results are reported in previous studies [14,20] and informed the planning of this study. In this study (Phase 2), we co-designed and developed the digital framework through workshops and prototype testing with various key stakeholders to address the needs identified in Phase 1. In Phase 3, the organizational requirements of the digital framework were tested for feasibility in clinical settings. These results further informed the design process. All phases were conducted iteratively throughout the PD study (see Figure 1). Literature studies were conducted continuously to broaden our understanding of the emerging findings. This paper presents and critically discusses the findings from Phase 2, which serves as a proof of concept for the digital framework.

### Ethical Considerations

PD research respects the fundamental human right to actively influence the design of digital technologies, elevating users from mere informants to recognized and integral participants in the co-design process [41]. To achieve this, a trustworthy and collaborative relationship among users, researchers, and technology designers must be established, providing users with the power to partake in decisions. Hence, all choices made by the design team and researchers were guided by user feedback through various user-engaging activities. Each user must willingly participate in such activities, working as themselves, with themselves, and for the task and project at hand [39]. All participants provided written informed consent and were informed that they could withdraw from the user activities at any time without consequences. The study was approved by the Danish Data Protection Agency (Journal 20/60035), and personal data were stored in compliance with the European General Data Protection Regulation (GDPR). To protect participants’ privacy and maintain confidentiality, data material was anonymized. The study was reviewed by the Regional Committees on Health Research Ethics of Southern Denmark and deemed exempt from the Danish Committee Act (case S-20252000‐37). Participants did not receive any compensation for their participation in the study.

### Setting and Participants

The setting was a surgical ward at Lillebaelt University Hospital, which provides treatment and care for acutely admitted adult patients primarily suffering from benign gastrointestinal disorders. The hospital is located in Southern Denmark, serving approximately 300,000 residents. The workshop participants included doctors, caretakers, patients, relatives, and a support team with skills in health care communication and quality, IT systems, information technology, and PD research. The health care providers were purposively selected to represent differences in gender, roles, seniority, and experience level in the surgical ward. Patients and relatives were enrolled during interviews conducted in the first phase of the study. Thus, in this study, these were former patients discharged within 1 to 2 months. In prototype testing, all eligible inpatients, relatives, and health care providers present were asked to participate. The inclusion criteria targeted acutely admitted Danish-speaking patients and their relatives who were ages 18 years or older. Individuals diagnosed with dementia, delirium, or other conditions leading to disorientation were excluded. Totally, 12 doctors were recruited, of whom 7 were highly experienced senior surgeons and 5 were junior doctors with low experience. The caretakers were either registered nurses or nurse assistants; some had special functions, for example, as specialist nurses, coordinating nurses, or head nurses. In total, 16 caretakers were recruited. A total of 13 patients and 9 relatives were recruited, and the support team consisted of 8 individuals. Altogether, 58 participants were enrolled in this second phase of the PD study (see Table 1).

**Table 1.** Characteristics of participants and their attendance in workshops and tests throughout the participatory design process.

Participants (n=58)	Characteristics		Overview of attendance, n				
	Males, n (%)	Experience^a^/age range	Creative workshop	Future workshop	Mock-up workshop	Laboratory testing	User testing
Doctors (n=12)	7 (58)	<0.5-20	5	5	2	0	9
Senior surgeons (n=7)	5 (71)	0.5-20	2	2	1	0	6
Junior doctors (n=5)	2 (40)	<0.5	3	3	1	0	3
Caretakers (n=16)	2 (13)	<0.5-21	4	4	1	0	13
Specialist nurses (n=2)	0 (0)	3-21	2	2	1	0	1
Work environment nurse (n=1)	1 (100)	2	1	1	0	0	0
General nurses (n=6)	0 (0)	<0.5-12	1	1	0	0	5
Coordinating nurses (n=2)	0 (0)	2-3	0	0	0	0	2
Head nurses (n=2)	0 (0)	<0.5-5	0	0	0	0	2
Nurse assistants (n=3)	1 (33)	1-11	0	0	0	0	3
Patients (n=13)	7 (54)	31-84	4	2	0	0	9
Discharged patients (n=4)	2 (50)	68-82	4	2	0	0	0
Inpatients (n=9)	5 (56)	31-84	N/A^b^	N/A	N/A	0	9
Relatives (n=9)	3 (33)	31-93	4	2	0	0	5
Partners (n=6)	2 (33)	59-93	3	2	0	0	3
Adult children (n=2)	1 (50)	39-50	1	0	0	0	1
Friend (n=1)	0 (0)	31	N/A	N/A	N/A	0	1
Support team (n=8)	2 (25)	0.5-15	4	6	4	5	5
Communications consultant (n=1)	0 (0)	5	0	1	0	0	1
Quality coordinator (n=1)	0 (0)	10	1	1	0	0	1
Technology designer (n=1)	1 (100)	14	0	0	1	1	0
IT-coordinators (n=2)	0 (0)	0.5-9.5	1	2	1	2	1
Robot technologist (n=1)	1 (100)	4.5	N/A	N/A	N/A	1	1
Researchers (n=2)	0 (0)	1.5-15	2	2	2	1	1

a Years of experience in the surgical ward/years of experience in current role.

b Not applicable.

### Data Collection

Data were collected through a series of workshops and prototype testing conducted between October 2021 and January 2023: (1) creative workshop generating ideas for the digital framework, (2) future workshop developing requirements needed to fulfill user needs, (3) mock-up workshop discussing initial design concepts, (4) laboratory testing of functionalities and user-flows of the initial prototypes, and (5) user testing of high-fidelity prototypes in clinical settings. The first 2 workshops were facilitated by 2 innovation consultants specialized in co-operative design processes, drawing on the concept of Future workshops developed by Jungk and Müllert [42]. These workshops were structured into distinct phases (critique, vision, and implementation) to collectively critique the current system and develop proposals for a more desirable future. The workshops were held in a conference room at the hospital and each lasted 4 hours. Data consisted of written post-it notes from participants, field notes taken by HP and JC, photographs, and audio-recorded transcripts. HP and the IT coordinators facilitated the mock-up workshop and the prototype testing. The mock-up workshop lasted 3 hours, while the laboratory and user testing spanned 46 hours over nine days. These activities were held in IT environments and real-life settings, respectively. Feedback reports with adjustments needed to ensure usability, along with photographs and screen prints, served as data for this part of the study. The user activities followed the PD approach, iterating through the steps: plan, act, observe, and reflect [40,41]. After each workshop or test, the researchers shared insights and perspectives as part of the initial analysis. Thus, each activity was planned based on reflections from the previous one, using detailed scripts outlining the various steps and responsibilities.

### Creative Workshop

The creative workshop focused on generating ideas for the digital framework based on user needs. A total of 21 team members participated in this workshop (see Table 1). The workshop comprised both a critique and a vision phase. In the critique phase, the participants were presented with the critical findings from Phase 1, allowing them to comment or contribute with new perspectives. In the vision phase, participants were divided into 4 groups and encouraged to list user needs and ideas to address them for each step of the SWR process: (1) during preparation, (2) in the patient room, and (3) when following up. A total of 2 groups entailed nurses and doctors, respectively, and 2 entailed a mix of patients and relatives. The support team was assigned to various groups, supporting the discussions, observing, and listening to the ideas and concepts being generated. Participants were encouraged to be creative and to record their thoughts, ideas, and visions without considering organizational or economic constraints. Each group recorded their needs and ideas on post-its and arranged them on posters illustrating the 3 steps of the SWR process. Posters were subsequently presented and discussed in a plenary session. After the workshop, the researchers and innovation consultants summarized the user needs and ideas into a Service Blueprint, visualizing the user journey of the SWRs.

### Future Workshop

The future workshop comprised the implementation phase, which aimed to develop feasible concepts based on the ideas generated in the creative workshop. A total of 19 team members participated in this workshop (see Table 1), which began with qualifying the Service Blueprint. The participants were divided into similar groups as in the creative workshop. First, the groups were asked to write supplementary comments or immediate ideas on post-its and place them on the Service Blueprint. Subsequently, each group was tasked with developing precise and realistic descriptions of requirements for selected ideas from the Service Blueprint. The final part was exclusively dedicated to the health care providers, who focused on developing a detailed organizational framework necessary for implementing the proposed technologies into clinical practice. Based on the workshop, product requirement specifications were developed by the research team, outlining prioritized requirements for the digital framework as specified by the users. The requirements specification process hinged on the idea that the users understood what the digital technologies should do and why, while the technology designers had the technical expertise to determine how to make it work. Thus, the requirements specifications were handed to an IT company for further processing. The specifications were not static and were constantly revised and refined through iterative processes and collaborations between users, researchers, and technology designers in the upcoming user activities.

### Mock-Up Workshop

Using the product requirements specifications as a starting point, 2 doctors, a specialist nurse, and 4 support team members participated in a mock-up workshop conducted at the IT company (see Table 1). During the workshop, participants created low-fidelity prototypes of the digital framework using simple, nondigital representations such as drawings and wireframes. The technology designers introduced various ideas for different design concepts through whiteboard sketches. This approach allowed the participants to explore multiple design directions through rapid and intuitive iterations before proceeding to more detailed design elements. From these sketches, initial wireframes of the digital framework were developed to agree on the basic structure and functionalities of the IT systems needed. The wireframes entailed visual representations of the basic idea of the digital framework. Following the workshop, the technology designers and IT coordinators created mock-up versions of the digital framework, which were handed to the health care providers and researchers for feedback and corrections. From these low-fidelity prototypes, a revised requirements document, and a specifications document describing detailed component requirements for the various subsystems of the digital framework were developed.

### Laboratory Testing

Based on the revised requirements documents, the IT-coordinators and technology designers developed high-fidelity prototypes of the IT systems. These prototypes were laboratory-tested by 5 members of the support team (see Table 1). In a test setup at the IT department, the prototypes’ performance, functionality, and security were tested in a controlled environment simulating real-life conditions without affecting live systems. The functionality of every single component was tested to verify whether the prototypes met the requirements and functioned correctly under various circumstances. Different usage scenarios were exposed to ensure the software handled the expected demands. Furthermore, compatibility was tested to ensure the software worked correctly across different devices (iOS, Android, and web). Feedback on requirements that were fulfilled or neglected was sent to the technology designers and IT coordinators to be refined or changed.

### User Testing

A total of 41 participants, including 22 health care providers, 14 patients and relatives, and 5 support team members, conducted user testing through simulated interactions with the revised high-fidelity prototypes (see Table 1). These versions closely resembled the look, feel, and functionality of the final products, and realistic data were used to replicate their actual use. Participants alternately tested the prototypes in a simulation room within the surgical ward. If patients could not move to the simulation room, the test setup was moved to their rooms. Each participant focused on testing the functionalities relevant to them while the researchers simulated the roles of the other participants. The purpose of the user tests was to ensure that the high-fidelity prototypes met user expectations and achieved precise adaptation in clinical practice, as well as to validate design decisions, visual aesthetics, and interactive elements. Detailed feedback on user experiences and interactions was sent to the technology designers and IT-coordinators for final revisions before releasing the advanced prototypes. Table 2 visualizes the various user-engaging activities and their outputs during the PD process.

**Table 2.** User-engaging activities and their outputs during the participatory design process.

User-engaging activities		Outputs (from user needs to advanced prototypes)
Workshops		
Creative workshop		Service Blueprint
Future workshop		Product requirements specifications
Mock-up workshop		Low-fidelity prototypes
Test setup		
Laboratory testing		Advanced prototypes
User testing		High-fidelity prototypes

### Data Analysis

Notes, transcribed material, and feedback gathered from each user activity were analyzed, inspired by systematic text condensation, to get an overview of each activity’s dominating themes, ideas, and feedback [43]. The analysis followed a 4-step process, beginning with a thorough reading of the text material while identifying preliminary themes (Step 1). Next, meaningful units from each data source were extracted (Step 2), organized into subcategories (Step 3), and grouped into broader overall categories (Step 4) [43]. Analysis matrices with direct quotes and post-it notes from participants, along with excerpts from the product requirements specifications, are provided in supplementary files to enhance the credibility and confirmability of the findings and design decisions.

## Results

### Service Blueprint

**Figure 2.**
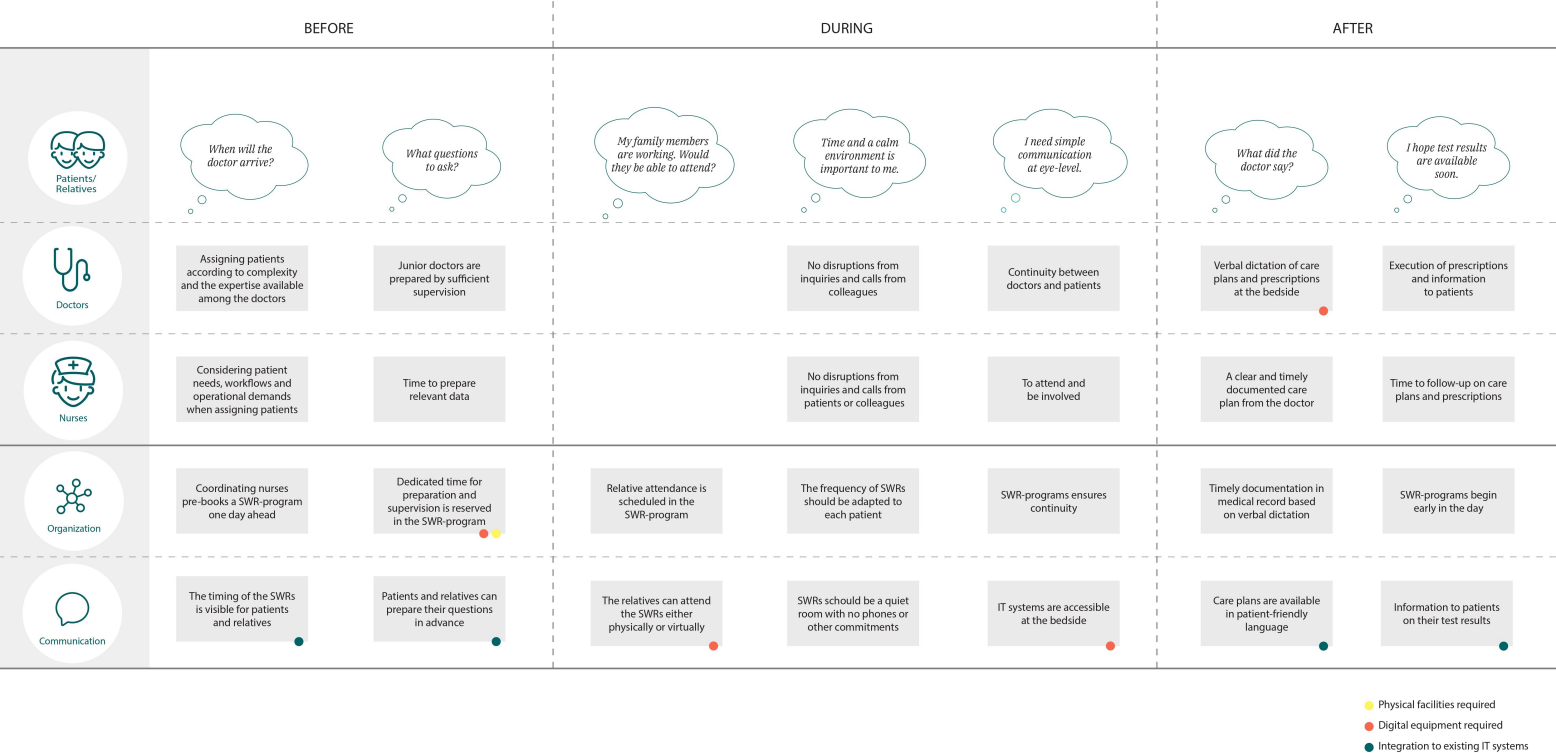
Service Blueprint visualizing dominating user needs across the surgical ward round (SWR) process (upper half) and suggested ideas to address them (lower half).

As a result of the creative workshop, the Service Blueprint (see Figure 2) mapped the structure and key elements of the SWR process, highlighting user needs and supporting processes. This provided an understanding of the relationships between the various steps of the SWR process, including the front-stage actions, back-stage processes, and IT systems needed to fulfill user needs. The Service Blueprint was vertically divided into three columns representing each step of the SWR process. Horizontally, the user needs of each group of participants were listed in the upper half section. In the lower half section, the back-stage organizational processes and front-stage communicative actions suggested to address user needs were listed. Dots represented demands for the physical facilities, digital equipment, and IT systems needed.

Dominating needs of patients and relatives were to be informed well in advance about the timing of the SWRs, allowing them to attend and prepare relevant questions. The doctors requested a more deliberate distribution of patients, considering the condition of patients and the expertise of the doctors. If the patient case were complex, junior doctors needed to be prepared through supervision from seniors. Nurses sought to have a say in the order of patients, considering patient needs, their workflows, and the operational demands of the ward when assigning patients. Furthermore, they required adequate time to prepare relevant patient data. Doctors emphasized that the nurses had the best overview of patients to properly distribute them and suggested that they should be responsible for planning a SWR-program. The nurses agreed but emphasized that the distribution process should not be too time-consuming for the individual nurse. Thus, it was decided that the coordinating nurses should be overall responsible for prebooking the SWRs a day ahead (see Multimedia Appendix 1). An important theme for patients and relatives during the SWRs was to have sufficient time in a calm environment to have an attentive conversation with health care providers communicating at eye-level. The health care providers wanted to minimize disruptions from inquiries and calls from other patients or colleagues during the SWRs by planning a dedicated time for the conversation. In addition, senior doctors suggested that the frequency of SWRs should be tailored to each patient and emphasized that continuity, achieved by conducting rounds on consecutive days with the same doctor, would lead to more efficient and attentive SWRs. Patients and relatives agreed that SWRs should be conducted only on days with a clear agenda. Furthermore, the participants agreed that IT systems should be available at the bedside to access relevant information and data. Patients and relatives highlighted that they appreciated when the health care providers visually displayed information from the electronic medical record on the computer screen, for example, test results, x-rays, or scans. Nurses emphasized that prescriptions and care plans should be handed directly to the care team at the bedside and be timely recorded in the medical record to ensure optimal follow-up. The doctors preferred to dictate their prescriptions verbally at the bedside to automatically integrate these into the electronic medical record, but needed updated systems and equipment to do that efficiently. Patients had difficulties remembering the information from SWRs. Thus, they requested access to verbal or written summaries of the care plans.

### Product Requirements Specifications

The product requirements specifications entailed the bottom lines of the Service Blueprint encompassing back-stage organizational processes and front-stage communicative actions to address user needs. These were expanded into more detailed requirement components, and the participants prioritized each from 1 to 3. The first priorities were “must-haves,” representing essential requirements. The second priorities were “should-haves,” representing requirements to be met if possible. The third priorities were “nice-to-haves,” representing nonessential requirements that were not critical to the core concept of the digital framework. Must-haves were a booking system to prebook the SWR-program, allowing the nurses to prioritize patients appropriately. Furthermore, the timing and names of the attending doctor and nurse should be visible to the patients and relatives. If possible, the timing should be presented as time slots with a defined start and end time. In addition, it was considered helpful, although not essential, if patients and relatives could access the agenda for the SWRs to prepare themselves by noting questions for the doctors. Furthermore, photo presentations of the health care providers were considered a nice-to-have feature (see Multimedia Appendix 1). Since computers-on-wheels with voice recorders were already available for health care providers to use at the bedside, and patients had access to their electronic medical records online to revisit care plans, developing new technologies to support communication during and after the SWRs was not a top priority. However, patients requested a more patient-friendly language in the electronic medical record.

### Low-Fidelity Prototypes

**Figure 3.**
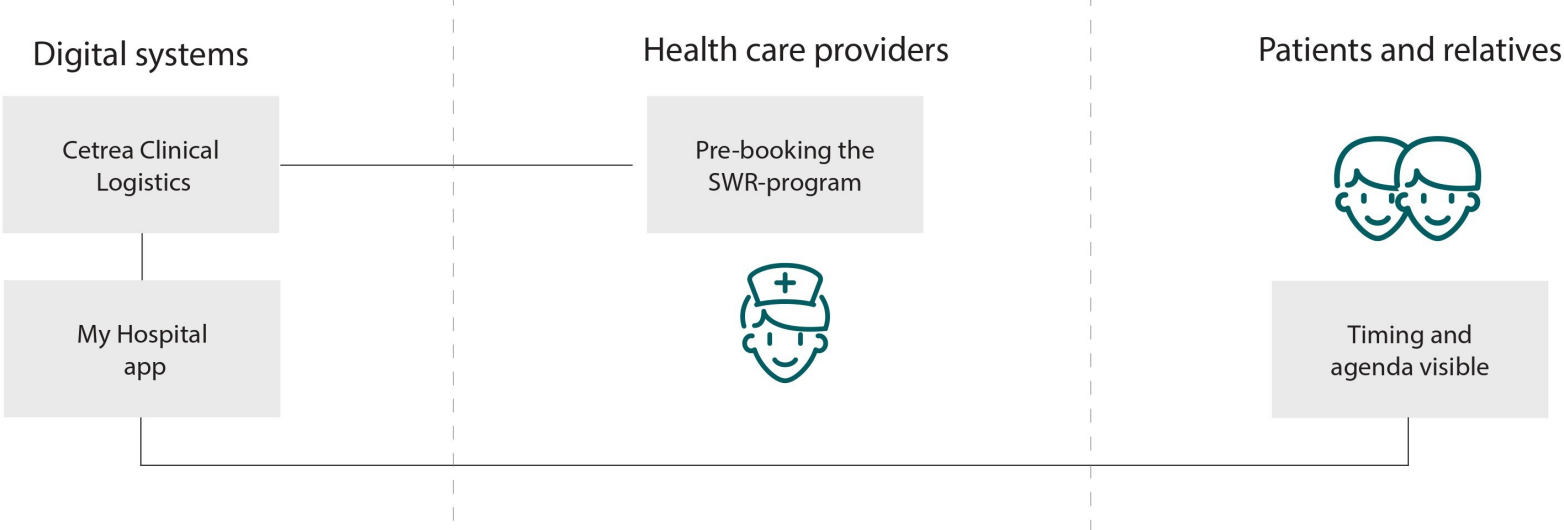
Wireframes of the basic idea of the IT systems to be part of the digital framework. SWR: surgical ward round.

The health care providers emphasized that automation and integration to existing IT systems were of utmost importance to ensure implementation of the digital framework. Thus, the initial wireframes entailed 2 central and integrated IT systems at the hospital (see Figure 3): (1) a logistics system used by the health care providers and (2) an mHealth app for patients and relatives. The health care providers suggested that the SWR-program should be developed as part of the existing IT system, Cetrea Clinical Logistics, which is the leading patient flow management solution in Denmark. The system was already in use at the department, providing an overview of central activities in the patient journey. To inform the patients and relatives of the SWR schedule, participants suggested that a module should be developed as part of the existing mHealth app My Hospital*,* used by patients across the Region of Southern Denmark. My Hospital was already integrated with the electronic medical record. However, to make data from Cetrea Clinical Logistics visible for patients, the technology designers proposed a software robot to enable automatic data transfer.

**Figure 4.**
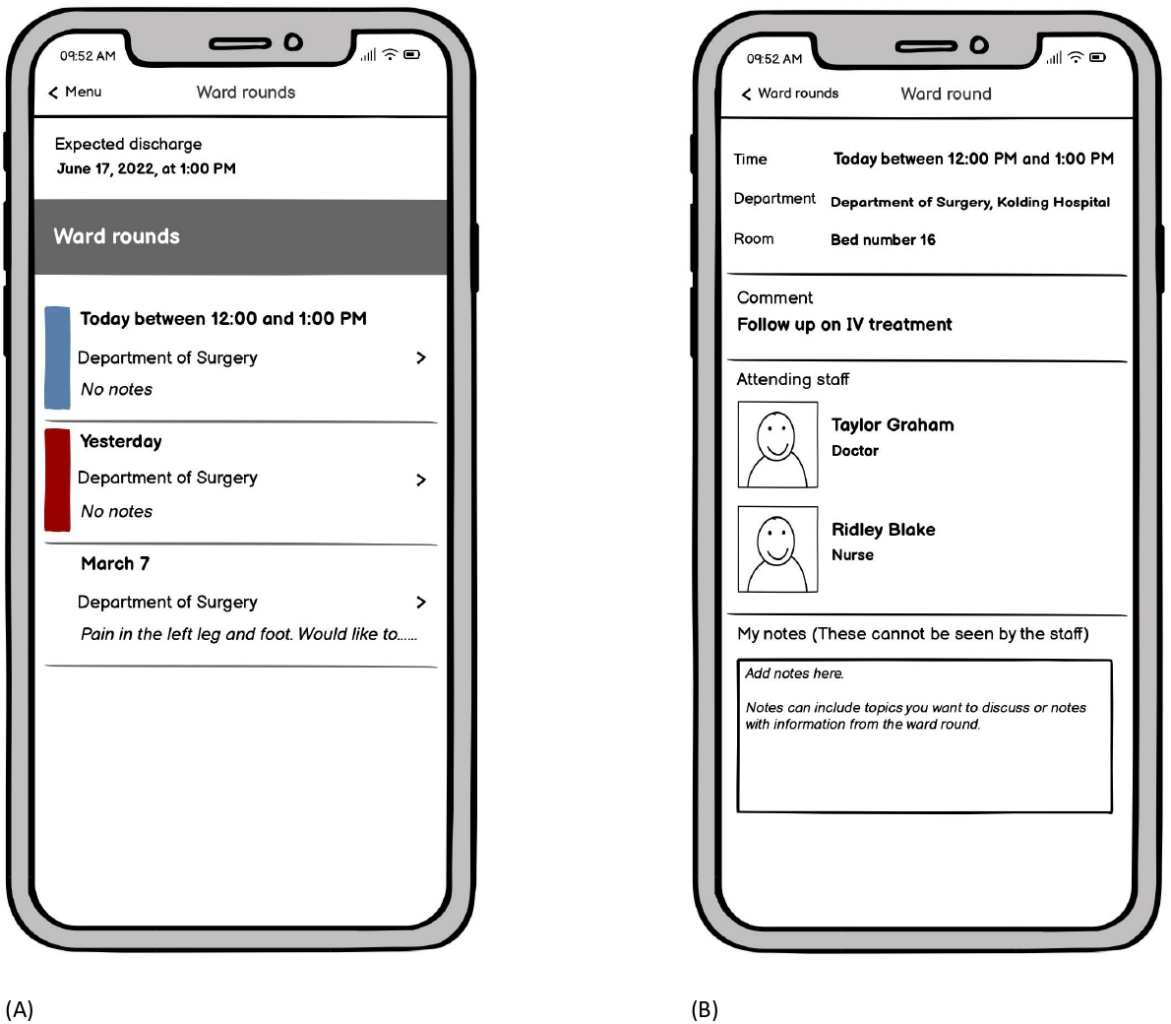
Mock-up version of the app module for patients and relatives.

The IT coordinators created the SWR-program in Cetrea Clinical Logistics, enabling the nurses to prebook the SWRs in time slots. To enhance interdisciplinary collaboration, names and diagnoses of patients, pictures, and telephone numbers of attending doctors and nurses, and the nurse agenda for the round appeared in the program. To make the timing and agenda visible to patients and relatives, the technology designers developed a mock-up version of the app module in My Hospital. A list of prebooked SWRs appeared in the first screen frame, along with the expected discharge date (see Figure 4A). To accommodate difficulties among patients in recognizing the SWR team, names and pictures of the participating doctor and nurse were provided in the second screen frame. In addition, a note section to prepare questions for the doctors was added (see Figure 4B). Using My Hospital as an IT platform enabled relatives to get access if the patient provided consent, and video communication was available.

### High-Fidelity Prototypes

Implementing the SWR-program required massive organizational changes. Thus, the logistics system was feasibility tested in clinical practice before proceeding (part of Phase 3). Using the SWR-program in clinical practice revealed a need for flexibility in time slots to be able to adhere to the appointments scheduled for visiting patients and the different workflows of senior and junior doctors. Thus, various widths of time slots and dedicated time for preparation, supervision, and follow-up were developed on individual SWR tracks. As senior doctors had multiple commitments and often prepared to visit 2 to 3 patients in a row, their time slots were set to 2 hours as the standard. Junior doctors generally prepared for one patient at a time. Thus, their time slots were set to 1 hour.

An emergency track was established for newly arrived or critically ill patients or patients who did not require a specific appointment. This track had no fixed time slots. Instead, the nurses prioritized the patients in order 1, 2, and 3 based on specific criteria. Ideally, a senior and junior doctor should manage this track collaboratively, freeing the doctors from the time-scheduled tracks from this commitment. To ensure attentive conversations and optimal use of time, it was decided that the health care providers should jointly agree with their patients on the timing of their next appointment at the end of each SWR. Nurses emphasized that the SWR-program should end at least an hour before shift change to ensure optimal follow-up. Once the SWR-program were fully developed in Cetrea Clinical Logistics, the robot technologist coded the data and shared it with the technology designers. Based on the available data, they developed a high-fidelity prototype of the app module. Laboratory testing led to multiple adjustments to ensure an interactive representation that appeared meaningful for patients and relatives. This version entailed the functionalities already agreed on in the mock-up version but featured realistic user experiences, making it suitable for user testing.

### Advanced Prototypes

**Figure 5.**
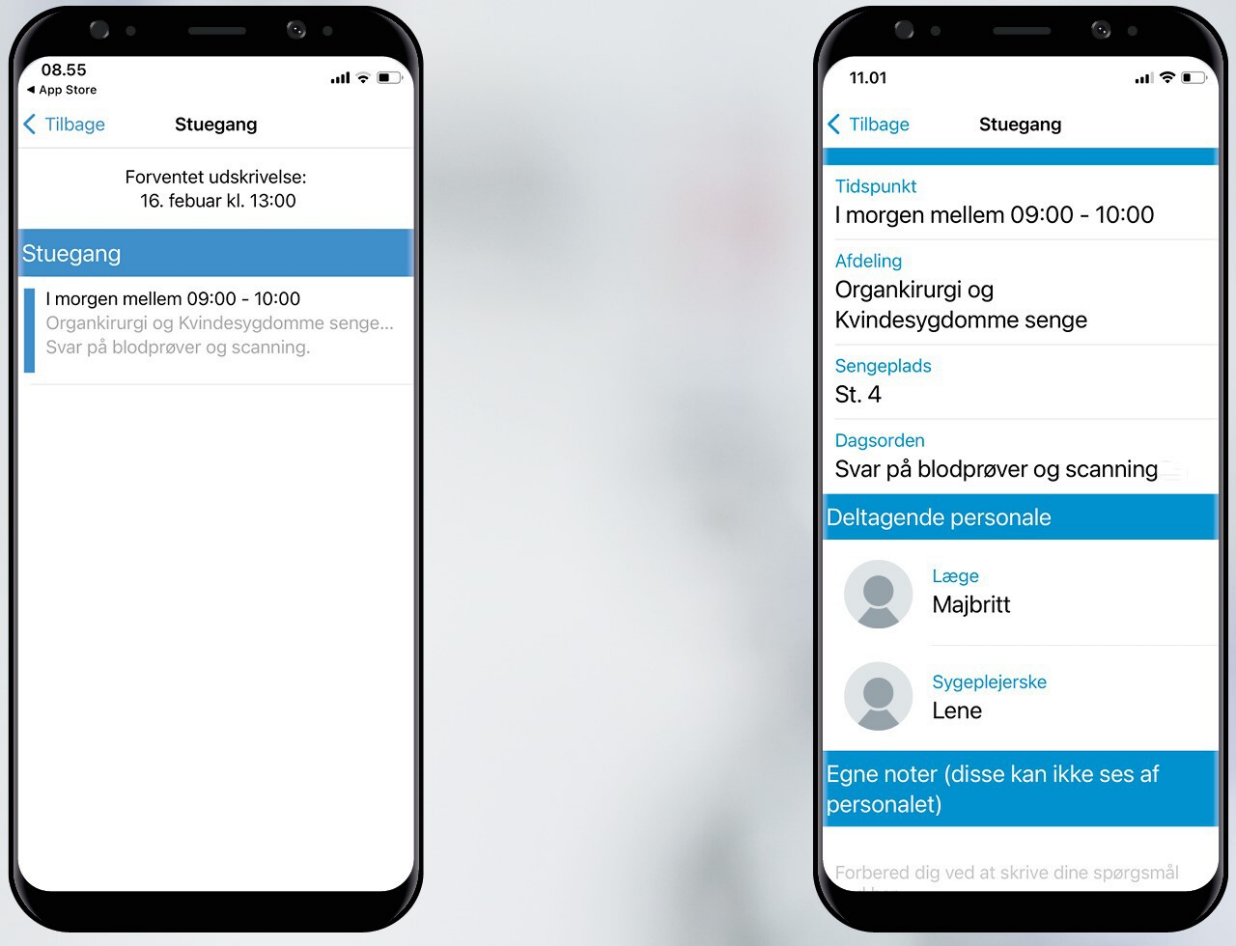
Screenshots of the advanced prototype of the mobile health app (Danish version).

**Figure 6.**
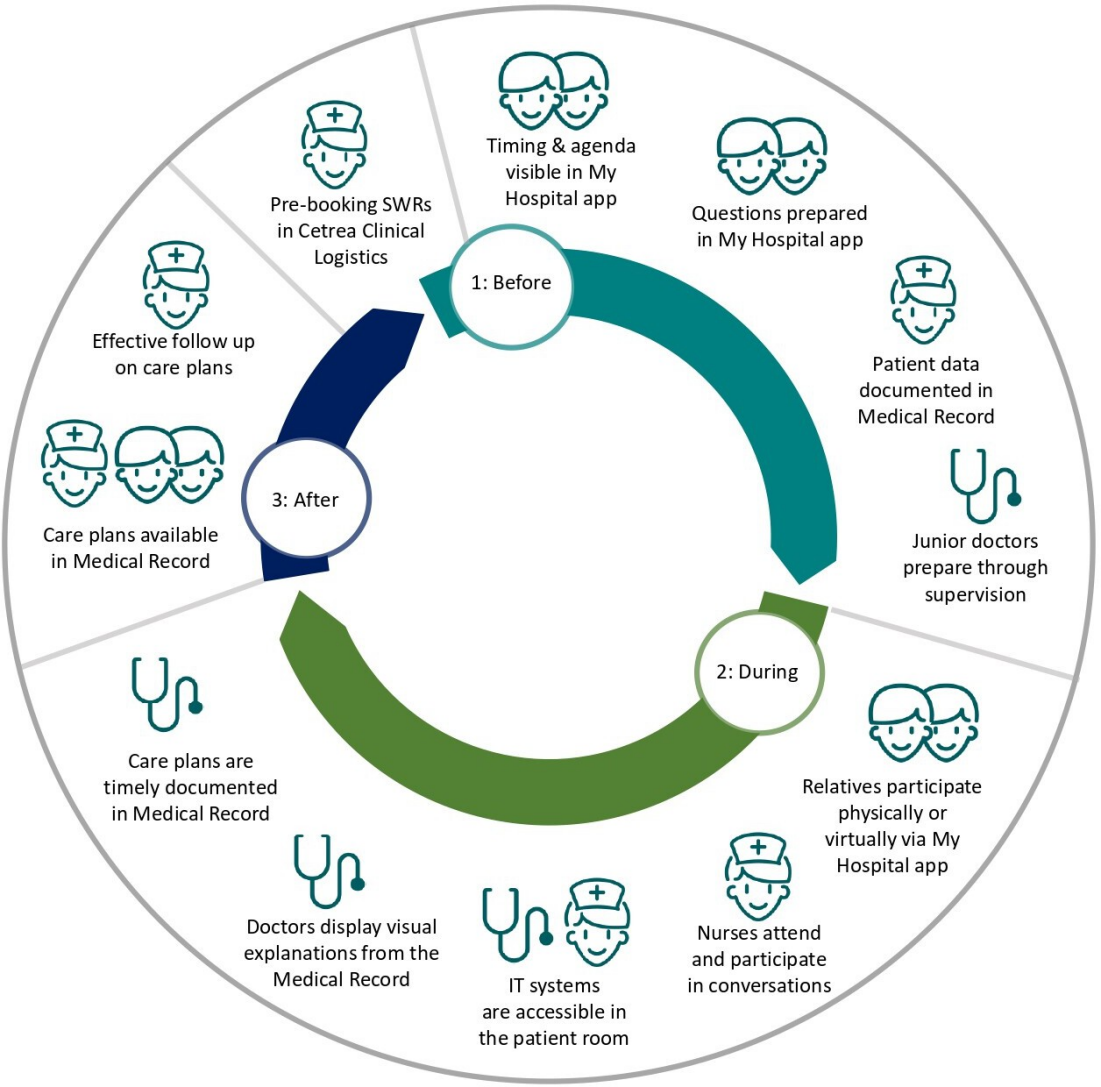
Digital framework to support a shared agenda at surgical ward rounds.

In the user testing, the caretakers requested that the SWR timing should be visible on their care lists along with other essential information about each patient. This functionality was added in Cetrea Clinical Logistics. Some health care providers reacted to their full names being displayed for patients in the mHealth app. However, from a patient’s perspective, knowing the names of the health care providers was desirable. Thus, surnames were removed, while first names remained. Some participants suggested that patients should be able to share their questions with the health care providers through the app. However, opinions on this were mixed. Some patients would like the health care providers to be prepared for their questions, while others preferred to keep their written questions private. Some doctors, especially the junior ones, would appreciate the chance to prepare for questions in advance, whereas others worried that they might not be able to fulfill the expectation of preparing for the questions beforehand. Some relatives expressed a wish to receive written responses to their questions in the app, especially if they were not able to attend the SWR. As only 1-way communication was technically possible in the high-fidelity prototype, transferring data from the app to the health care providers was not feasible. Thus, the preparation of questions remained a private matter. Some patients, particularly the elderly and frail, had limited digital health literacy and required assistance from caregivers to use the mHealth app. To address this, simple user manuals were developed, and iPads were made available for patients who wanted to use the app but did not have a device. If patients were still unable or unwilling to use the app, the users suggested that the information should be provided in an analogue format on whiteboards at the bedside. Due to ongoing adjustments of the SWR-program during the day, the health care providers noticed a risk of spamming patients with incorrect bookings if the software robot operated continuously. Participants agreed that the highest priority was to avoid confusing patients with frequent changes. Therefore, they decided that the robot should be activated at scheduled times: at 2:30 PM, once the SWR-program for the next day was planned, and at 9:00 AM, when the doctors and nurses had entered their names into the program. Yet, this decision did not allow electronic notifications to be sent to patients about potential delays in the SWR-program, which was a major concern for the health care providers. To align expectations with the patients and relatives, they were informed that time slots were estimated and delays might occur, which they fully accepted. Yet, nurses reiterated the need for improved adherence to the time slots, especially among the senior doctors. Senior doctors expressed a desire to know when relatives attended the SWRs, allowing them to be even more mindful of time slots in those cases. To support this, it was agreed that nurses should note in the SWR-program whenever relatives were present (see Multimedia Appendix 1). Furthermore, a steering committee, comprising 2 specialist doctors, the department management, and a clinical nurse specialist, was appointed overall responsible for potential further adjustments of the SWR-program during the forthcoming implementation process. Ultimately, advanced prototypes of the logistics system and the mHealth app were released (see Figure 5). These, along with the electronic medical record, constituted the digital framework developed through the PD process (see Figure 6).

## Discussion

### Principal Findings

Using PD, we collaboratively designed, refined, and tested a unified and context-sensitive digital framework to support a shared agenda at SWRs. The highest priority of the users was to improve the processes leading up to the SWRs, and they emphasized that the presence and readiness of all core participants was essential for initiating a person-centered dialogue. To facilitate this, a logistics system was developed, enabling the coordinating nurses to prebook SWRs a day ahead and allowing patients and their relatives to access the schedule through an mHealth app. The design of the digital framework was guided by the assumption that increased transparency around the timing and content of SWRs, combined with the opportunity for patients and families to submit questions in advance, could enhance their sense of preparedness and support more active engagement during the round. Although the framework primarily targets logistics, it represents an initial step toward reshaping the nature of ward round conversations from being predominantly doctor-led to being more collaborative and person-centered. Workshops and prototype testing played a crucial role in developing the digital framework, enabling ongoing refinement in close collaboration with users until an acceptable and contextually appropriate solution was achieved. Thus, our study, like many others [44], underscores the significance of the active collaboration between technology designers and users as a key to developing innovative digital technologies that can be successfully integrated into the health care system. More specifically, our study demonstrates how PD can be used to navigate technical and organizational constraints that might otherwise hinder implementation.

### Comparison With Previous Work

Providing the participants with a solid foundation for preparation adheres to the core principles of initiating a person-centered dialogue. According to the Calgary-Cambridge guide for evidence-based health care communication, a key aspect of initiating the encounter is to confirm the issues to be discussed and to screen for additional questions, thereby negotiating a shared agenda for the encounter [22,23]. This process ensures that both the agendas of the health care providers and those of the patients and relatives are incorporated into the dialogue. The digital framework aims to support this, by facilitating patients and relatives to prepare themselves by documenting their questions in the mHealth app beforehand. Correspondently, Walton et al [45] suggest that preparing patients for what to expect and providing them with the round schedule might facilitate their inclusion in conversations and lead to more person-centered communication. Furthermore, in video-consulted rounds with relatives [38], patients describe the benefit of having a fixed time, allowing them to prepare in advance.

At our hospital, basic communication behaviors are taught through communication skills training based on the Calgary-Cambridge guide. This training has shown positive effects on the health care providers’ self-efficacy and communication behavior, fostering a more person-centered approach [46,47]. Nevertheless, our study emphasizes the importance of considering the organizational frameworks that shape the encounters, particularly in the wards where key participants may be absent or unprepared to engage in the dialogue. Several studies [34-38] suggest that enabling video communication can offer family members flexible alternatives to participate and enhance their involvement in patient care. However, most family members perceive video calls as a supplementary option and prefer in-person communication, especially when conversations include serious messages [35,36]. Furthermore, time, culture, and change of work routines have been found to be the primary barriers to implementing video communication [37]. The digital framework developed in this study supports the organizational changes necessary to coordinate family participation at SWRs, with video communication as an option when physical presence is not feasible.

Another essential yet often overlooked behavior of health care providers is to begin the encounter by greeting the patient and introducing themselves and their roles [22,23]. The mHealth app supports this by providing names and pictures of the attending doctor and nurse for patients and relatives to recognize the SWR team. Similarly, other studies [31,33] have reported high satisfaction levels and perceived usefulness of apps delivering patient information, along with pictures, names, and role descriptions of care team members. Vawdrey et al [33] noted that patients regarded care team information as one of the most beneficial features. In addition, O’Leary et al [31] found that providing this information significantly increased the percentage of patients recognizing their attending doctor. Nevertheless, these apps proved not to affect patient activation.

Investigating interdisciplinary collaboration, Walton et al [48] emphasized that having the right individuals present at the right time, along with a clear understanding of each person’s roles and responsibilities, is essential for effective teamwork. In addition, several studies [26-28] indicate that advance notifications of round schedules increases nurse attendance, fosters cultural change, and may ultimately improve patient outcomes, including greater satisfaction, improved care coordination, and slight reductions in length-of-stay. The digital framework, developed in our study, went even further and gave the nurses the power to influence the SWR schedule. This represents a significant shift from the traditional round culture, in which the doctors solely dictated the timing and agenda for the SWRs. The nurse agenda was clearly outlined in the SWR-program to be integrated into the discussions, as recommended in the Calgary-Cambridge guide [22,23]. Correspondently, Truelove et al [29] identified that nursing-centered round schedules and including nursing input at the beginning of encounters were critical factors for improving nurse attendance. Furthermore, the nurse agenda was visible for patients and relatives in the mHealth app. Accordingly, Vestergaard et al [36] suggest that predefining the topic of rounds might help family members to attend to important messages. However, future versions of the mHealth app should consider allowing patients and relatives to influence the round schedule and share their questions with health care providers in advance. Similarly, Ratelle et al [49] suggest that encouraging patients to inform health care providers about their goals, concerns, and questions might prepare doctors to address these issues and consider psychosocial factors extending beyond the hospital stay.

Although the process leading up to the SWRs was the primary focus area of the digital framework, the users emphasized several essential aspects to consider during and after the SWRs. These include minimizing interruptions, communicating at eye level, providing tailored explanations and illustrations, and clarifying care plans and next steps. Each of these practices are central aspects of evidence-based health care communication [22,23] and the digital framework support them in various ways. Scheduling the SWRs might reduce interruptions and foster more attentive dialogues. Furthermore, bringing IT systems to the bedside allows health care providers to access visual illustrations and information from the electronic medical record, dictate mutually acceptable care plans at the bedside, and collaboratively schedule the next SWR. The use of mobile devices such as tablets or computers-on-wheels for information sharing and patient engagement during rounds has been investigated in several other studies [50-52]. Crowson et al [52] found that the use of mobile tablets significantly shortened the round duration and increased time spent with patients. This suggests that mobile devices can effectively reduce time-consuming activities, such as leaving the bedside to look up medical queries and ease documentation practice. However, the extent to which doctors use these mobile devices varies significantly [50,51]. Future studies should investigate acceptable and time-efficient approaches, such as ambient artificial intelligence [53], to enhance bedside rounding documentation to foster more attentive conversations, provide patient-engaging information, and optimize follow-up care.

Engaging the health care providers in developing and testing the back-stage organizational processes of the digital framework proved vital for ensuring feasibility and minimizing the risk of resistance to use the IT systems. By addressing user needs from the outset, our study demonstrates how digital systems can be tailored to meet the expectations of all user groups, including health care providers, patients, and their families. Actively involving the users not only kept our focus on user needs but also revealed how integrations to existing IT systems as well as the clinical workflows of the health care providers needed to be addressed to successfully integrate the digital framework into clinical settings. Correspondently, Esdar et al [32] revealed that the adoption of mobile IT solutions was associated with close user participation and organizational cultures of innovation. Similarly, Andersen et al [54] highlighted that for mHealth prototypes to be successful, it was crucial to align or reconcile the concerns of patients and relatives with those of the health care providers, ensuring that both perspectives are considered and addressed. Failure to do so may lead to reluctance to use the prototypes. The user-engaging activities conducted in this study enabled us to develop a feasible solution for all stakeholders. In this way, our study refines current understandings of how structured SWRs should be designed to meet the demands of real-world clinical environments. Flexibility proved essential, allowing the digital framework to be adapted to the clinical context of the study. These findings provide valuable insights for the development of future collaborative digital solutions in health care, emphasizing the need for continuous engagement with key stakeholders and the flexibility to accommodate diverse needs.

### Limitations

In PD studies, the user-engaging activities typically involve all key stakeholders throughout the process [40]. In our study however, it was not possible for patients and relatives to attend the mock-up workshop at the IT company, and only 3 health care providers participated in this activity. To ensure their voices were genuinely heard, a large group of health care providers, patients, and relatives (n=36) took part in the user testing, offering invaluable feedback on the final design.

As recommended in PD, the researchers should remain flexible and open to various user suggestions [40]. While we strived to maintain this approach, limitations in resources meant we could not address every user request. Future studies should explore ways to integrate more interactive elements into the digital framework, as suggested in the user testing. The study was conducted at a single clinical site, which may limit transferability of the findings. However, the PD process was informed by insights from previous research, which helped integrate the perspectives and needs of a diverse patient population and a wide range of experienced health care providers. While certain aspects of the framework, such as the focus on logistics, patient and family engagement, as well as the use of digital technologies to facilitate collaboration, are likely to be applicable in other acute and surgical health care settings, some elements, such as specific workflows and institutional norms at our study site, may be more context-dependent. Further research in different health care settings is essential to assess transferability of the digital framework and refine its applicability across various contexts. Furthermore, as the study is currently at the proof-of-concept stage, the digital framework requires further validation and testing to establish its effectiveness in achieving real-world quality improvement outcomes. Although the digital framework was developed to support the preparation of patients and families for SWRs, its actual impact on enhancing their readiness and participation was not evaluated in this study. Additional research is needed to assess how the digital framework influences patient and family preparedness, as well as their engagement in SWRs.

### Conclusions

The PD process led to the development of a unified digital framework to support person-centered communication at SWRs, including a logistics system for nurses to prebook SWRs in designated time slots, making the schedule visible to patients and relatives via an mHealth app. Engaging key participants in the design and development helped uncover technical and organizational constraints that must be addressed to successfully integrate the digital framework into clinical contexts, while preserving its value for patients and their families. In conclusion, our study offers important insights by demonstrating how PD can be used to adapt digital technologies, ensuring they are both user-centered and context-sensitive. The next step of the research aims to pilot-test the digital framework in clinical settings and explore whether it fulfills its purpose of securing broader participation in SWRs.

## Supplementary material

10.2196/69679 Multimedia Appendix 1 Data material from workshops and user-engaging activities.

## Abbreviations

GDPR: General Data Protection Regulation
mHealth: mobile health
PD: participatory design
SWR: surgical ward round
