# Supplementary material for: Proof of Concept for a Digital Framework to Support a Shared Agenda at Surgical Ward Rounds: Participatory Design Study
Source: J Particip Med. 2025 Jun 19;17:e69679. doi: 10.2196/69679 (PMC12199840; doi:10.2196/69679)
Supplement: Multimedia Appendix 1 [file jopm-v17-e69679-s001.docx]

**Supplementary file – Data material from workshops and user-engaging activities**

**Creative workshop**

Analysis matrix condensing user needs and participant ideas derived from post-it notes

| **Meaningful units**  **Post-it ideas (Examples)** | **Subcategories**  **User needs** | **Overall category:**  **Before the rounds** |
| --- | --- | --- |
| SMS/email/notification as early as possible with the scheduled time.  Booking of an appointment, similar to scheduling a time at the outpatient clinic.  Ward rounds only on days with a clear agenda. | Timing of the rounds | Patients and relatives |
| To be informed of the names of the doctor and nurse. | Names of the participants |  |
| Note down your questions and own agenda. | To be prepared |  |
| Prioritization, ideally by the nurse, with predefined times based on the patient's condition and required examinations.  Avoid ward rounds for all patients every day. | Prioritization of patients | Doctors |
| Ward rounds in specialist teams, senior and junior doctors together.  Supervisor role, fewer patients, and more availability.  Scheduled supervision.  Office facilities and telephones available. | Supervision of junior doctors |  |
| Reallocation of tasks.  To know the timing of the ward round and which doctors is assigned.  Scheduled time for a dialogue with the doctor.  Physical facilities and telephones available. | Time to prepare | Nurses |
| Coordinating nurses manage the allocation and prioritization ensuring the right doctor is assigned to the right patient.  Structured and efficient, based on a plan or template.  Like an outpatient plan.  Ward rounds early in the day. | Influence on prioritization |  |

**Future workshop**

Product requirement specifications as a result of the needs and ideas provided by the users.

**User needs**
Patients and their relatives would like to know the schedule and attendees of the ward rounds, so they can be prepared for the discussion. Doctors and nurses need a more efficient organization of the ward rounds, with clear prioritization of patients, ensuring that each one is seen by the appropriate doctor in the correct order.

| **Overall category:**  **Organization** | **Meaningful units**  **Description** | **Priority** |
| --- | --- | --- |
| **Sub categories:**  Coordinating nurses | The coordinating nurses schedule the ward rounds based on patients' condition and needs, doctor competencies, and the department's workflow (preferably the day before, by 2 PM). | 1 |
| **Communication** | **Description** | **Priority** |
| Timing | Patients and their relatives are informed of the scheduled time for the ward rounds, possibly presented as time slots with a defined start and end time. | 1  2 |
| Participants | Patients and their relatives are informed about which doctor and nurse will be conducting the ward round,  possibly accompanied by a photo presentation | 1  3 |
| Agenda | Patients and their relatives are informed about the agenda for the ward round meeting. | 3 |
| **’System integration** | **Description** | **Priority** |
| Cetrea Clinical Logistics  Electronic Medical Record | Integration with Cetrea/Medical record is established, allowing staff to use their existing systems for booking patients, with a notification automatically sent to the patients. | 2 |

1 = Must have: Essential requirements
2 = Should have: Requirements to be met if possible
3 = Nice to have: Requirements not critical to the core concept

**User testing**

Analysis matrix condensing user feedback on adjustments suggested to the high-fidelity prototypes.

| **Meaningful units**  **Quotes (examples)** | **Sub categories** | **Overall category** |
| --- | --- | --- |
| It would be helpful if the scheduled time were included on our care lists. It is the first thing I note down in the morning. This would save me at least five minutes each morning, as I would not have to check if and when my patients are booked (Nurse) | Timing visible on care lists | Timing and attendees |
| She (the patient) asks first thing in the morning who will be coming for the ward round (Female relative, 31 years old)  Should my full name be listed there? (Senior doctor)  There are a few patients you would prefer to keep at arm's length (when it comes to sharing your last name). Luckily, we do not have many of them (Senior doctor) | Names of the healthcare providers |  |
| The only thing missing is a button labeled 'share questions with staff.' Often, they come in, say, *“I’m not sure, I’ll need to check with a colleague,”* and then leave. It would be really helpful if they were prepared for my questions (Female patient, 33 years)  It is useful to prepare for the questions before going in, so you are ready and not caught off guard. While we, as junior doctors, can handle many things, it may require us to look them up first (Junior doctor) | Sharing questions in advance would prepare the (junior) doctors | Interactive elements |
| It is a challenge if relatives have noted questions (in the mHealth app) but cannot attend the ward round, as the staff is unable to see and respond to them (Male relative, 50 years old) | Some relatives request a response to their questions in the mHealth app |  |
| I think its fine that they cannot see my notes and questions. I write them down in my own notes whenever I have questions, as otherwise, I might forget them (Female patient, 31 years old)  I think they should keep it as their own notebook (Senior doctor) | Private notes is preferred by some patients and doctors |  |
| I am concerned that if they can share it, there will be an expectation that we have seen it. If they are able to share it with us, we should also be able to manage it (Senior doctor)  There should be a limit, otherwise some people might write 27 questions. You could set a limit of 100 characters at most (senior doctor) | Patient expectations may not  be met |  |
| Those without an iPad should be assisted by a caretaker. It is undignified to repeatedly tell the patient, *"you need to use your iPad."* Those who are unable to do so should receive proper guidance (Female relative, 80 years old) | Assistance from caregivers | Digital health literacy |
| I just need an introduction to it. Then I am confident I will be able to assist the patients (Nurse assistant) | User manuals are needed |  |
| We do not have an iPad or a smartphone (Male patient, 76 years old)  IPads should be available for patients (Nurse) | IPads should be available |  |
| Bedside whiteboards would be better suited for the older generation (Male relative, 50 years old) | Analogue alternatives for non-digitals |  |
| Is it possible that I end up spamming four patients if I want to notify one patient about a delay? (Senior doctor)  I think they should be given a scheduled time without being notified of delays. If a delay occurs, one can just pop in and inform the patient that there is a slight delay. Giving multiple times might confuse some patients more than it helps (Nurse)  You can easily end up sending double bookings if you run the robot multiple times a day (Nurse) | Risk of spamming patients in case of delays | Managing the software robot |
| It makes sense for the coordinating nurses to run the robot (at specific times) to ensure the correct times are sent out to the patients (Coordinating nurse) | Activating the software robot |  |
| It requires that the doctors are held accountable for adhering to the schedule (Nurse)  We should inform the patients that we will try to adhere to the time slots, though we cannot guarantee it (Senior doctor)  Its fine to wait for an hour, but sitting for several hours without being given a time – I do not think that is acceptable (Male relative, 50 years old) | Adherence to time slot structure | Adherence to schedule |
| It would be a good idea to note in the system if we know a relative will be joining (Nurse) | Relative attendance |  |
